# Neurofibromatosis Type 1-Associated Plexiform Neurofibromas of the Face and Adjacent Head Regions: Topography of Lesions and Surgical Treatment Data of 179 Patients

**DOI:** 10.1007/s12663-022-01838-8

**Published:** 2023-01-19

**Authors:** Reinhard E. Friedrich, Manuel Modemann

**Affiliations:** Department of Oral and Craniomaxillofacial Surgery, Eppendorf University Hospital, University of Hamburg, Martinistraße 52, 20246 Hamburg, Germany

**Keywords:** Neurofibromatosis type 1, Plexiform neurofibroma, Surgery, Heat map

## Abstract

**Introduction:**

Facial plexiform neurofibromas (FPNF) are rare tumors frequently diagnosed in patients with neurofibromatosis type 1 (NF1), a tumor predisposition syndrome. FPNF often grows invasively and destructively, which may complicate surgical treatment. Data on the frequency, location, and surgical procedures of patients with NF1-associated FPNF are scarce. This study provides treatment data from a nationally networked reference center for the treatment of NF1 patients.

**Material and Methods:**

The localization and treatment data of 179 NF1 patients with FPNF were analyzed. Photographically documented tumors of the study area, further determined by imaging, were manually transferred to a facial scheme and digitized. The digitized registrations of the facial extensions of the tumors of each patient were overlaid in a single image (Photoshop™), so that the file of the facial scheme contained the sum of the tumor localization. Finally, the frequency of tumor localization was indicated with a color code. The frequency of tumor extension-related coded colors was applied to outline the lesions' topography on schematic face drawings (heat map).

**Results:**

The distribution of the tumors showed no side preference. The need for the treatment of patients with orbital/periorbital manifestations became evident in the graphic representations. Tumors do not respect anatomical units. However, the classification of the face according to dermatomes, especially the trigeminal nerve, offers indications of tumor spread and guides treatment planning. The mean number of surgical measures per patient was 2.21 (median: 1). Extensive swelling, hematoma, and delayed wound healing were all common postoperative complications.

**Conclusion:**

The color-coded, schematic overview of the frequency distribution of cutaneous tumor spread in NF1 patients with FPNF illustrates the importance of orbital/periorbital and cheek tumor manifestations in patients' treatment needs. The imaging procedure is suitable for controlling natural tumor growth in the same way as the documentation of the post-surgical course. Repeated interventions in the region are included in surgical planning of the progressing tumor disease.

## Introduction

Neurofibromatosis type 1 (NF1) is a monogenic, autosomal-dominant inherited tumor predisposition syndrome. The causative gene (17q11.2) encodes a protein (neurofibromin) with tumor suppressor characteristics. Multiple peripheral nerve sheath tumors (PNST) called neurofibromas are pathognomonic for NF1. The penetrance of the syndrome is almost complete in affected individuals. However, the phenotype of NF1 patients is highly variable.

The NF1-associated PNST arises from Schwann cells or their precursors. From a clinical point of view, two distinctions in NF1-associated PNST are relevant: cutaneous and plexiform. Facial plexiform neurofibroma (FPNF) can cause significant aesthetic disfigurement and functional impairment. Data on the frequency of FPNF are uncertain because the records are based on different sources or evaluation criteria [1, 2]. Among the body regions, craniofacial manifestations of PNF are frequently detected [3].

Surgical treatment options are limited and focus on tumor reduction, osteotomies, face lifting, and especially measures improving visual pathway clearance in orbital-periorbital manifestations of the lesion [4, 5]. An orienting classification of the facial tumor spread can be made by assigning the lesions to the cutaneous territories of the craniofacial nerve, in particular the trigeminal [6, 7]. However, tumor growth often exceeds the territorial boundaries of a nerve branch [8]. A visible FPNF, on the other hand, may not have infiltrated the entire cutaneous territory of the affected nerve branch(es) [9, 10]. This study determines the extent of facial tumors and surgical procedures. The objectives of the study were to evaluate clinically relevant treatment data of patients with FPNF and to determine the frequency distribution of these tumors in the study area.

## Material and Methods

### Patients

**Fig. 1:**
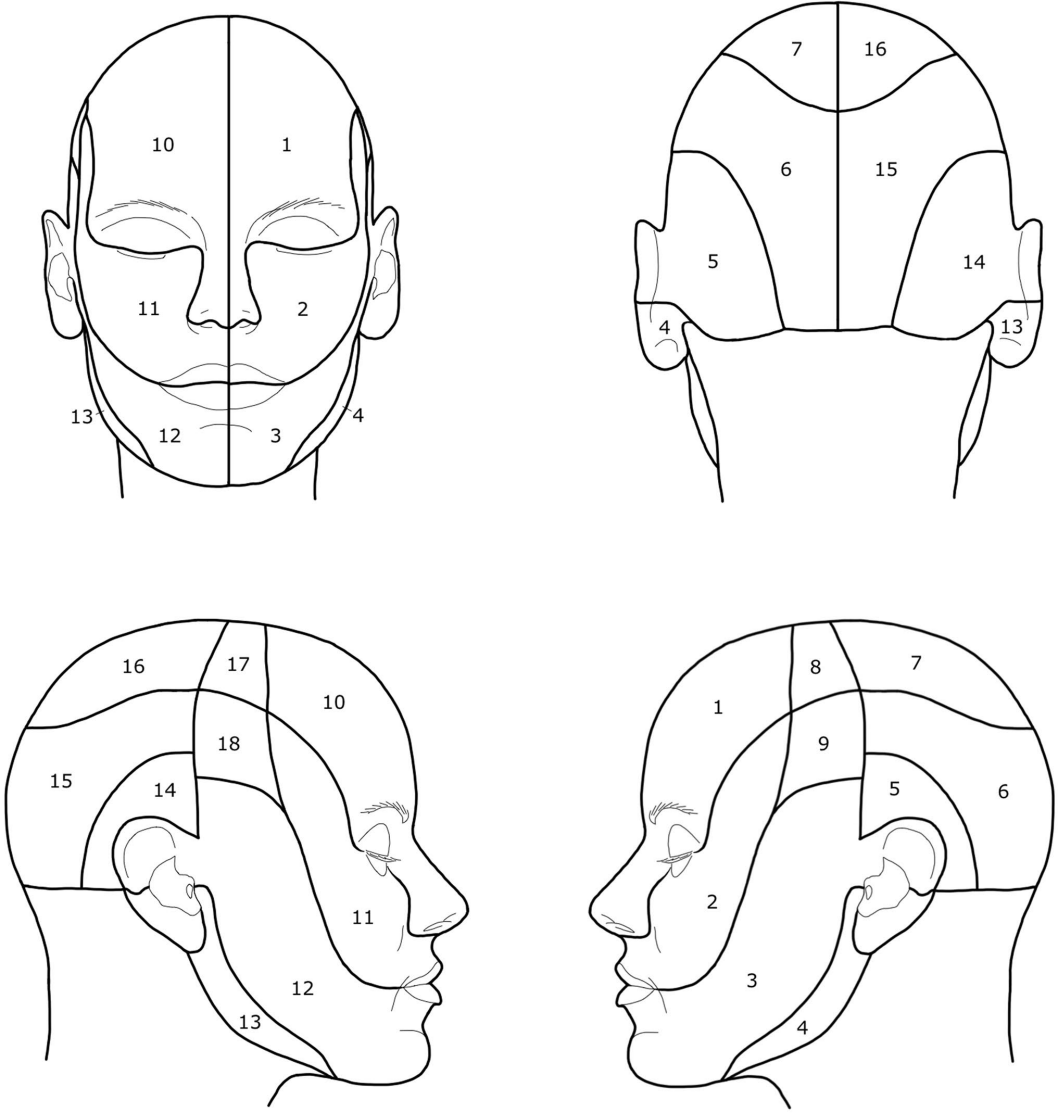
A figure series depicting dermatomes of the head region. The white lines show the division of the head based on the dermatomes. 1 and 10: N. ophthalmicus (N. V1), 2 and 11: N. maxillaris (N. V2), 3 and 12: N. mandibularis (N. V3), 4 and 13: N. auricularis magnus (C2, C3), 5 and 14: N. occipitalis minor (C2, C3), 6 and 15: N. occipitalis major (C2), 7 and 16: N. occipitalis major (C2)/N. ophthalmicus (N. V1), 8 and 17: N. mandibularis (N. V3)/N. ophthalmicus (N. V1), 9 and 18: N. mandibularis (N. V3)/N. maxillaris (N. V2) (according to Radlanski and Wesker (12), modified)

**Fig. 2:**
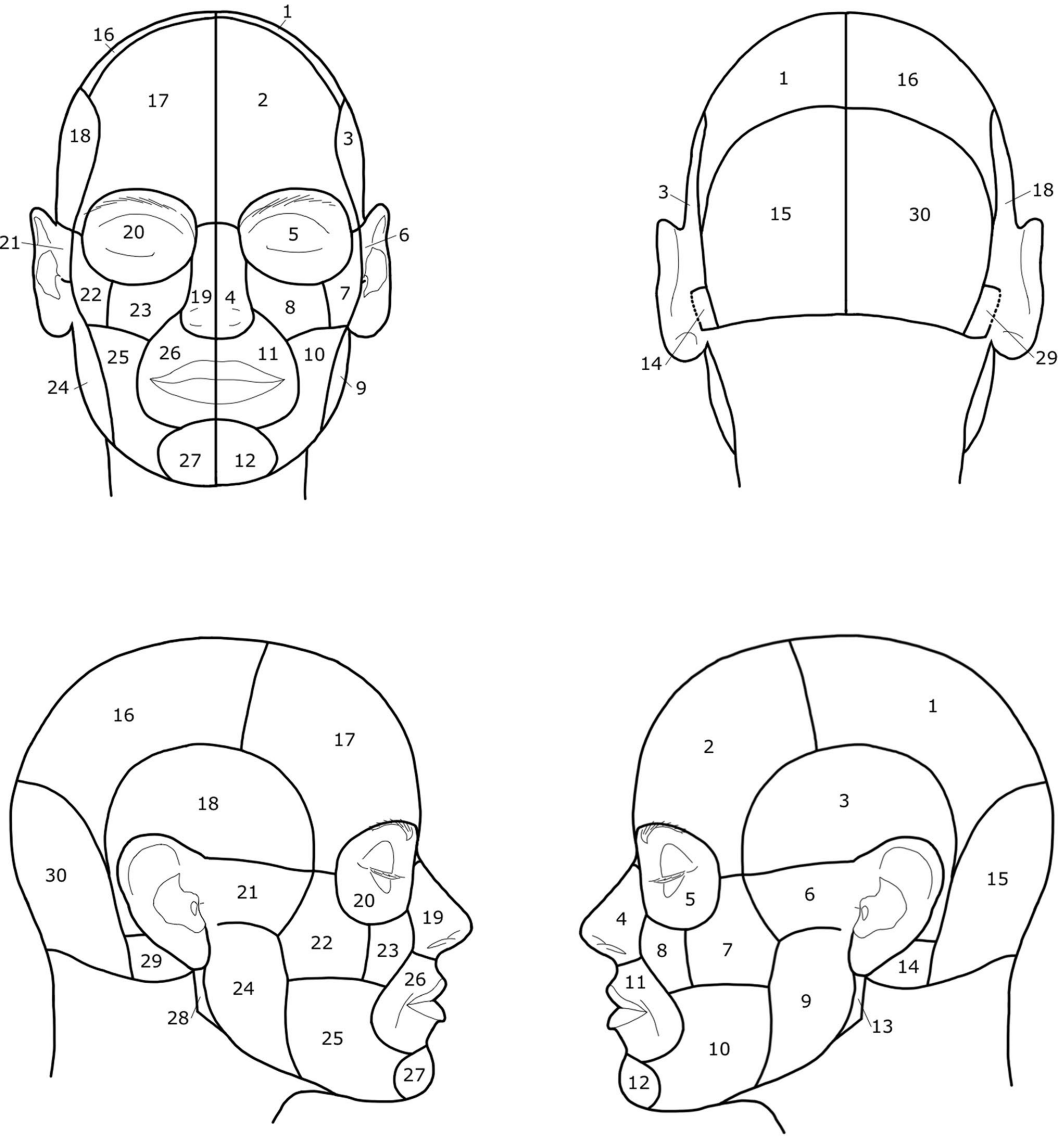
A series of figures illustrating anatomical units of the surface of the head region. The white lines show the division of the head based on the anatomical units. 1 and 16: Regio parietalis, 2 and 17: Regio frontalis, 3 and 18: Regio temporalis, 4 and 19: Regio nasalis, 5 and 20: Regio orbitalis, 6 and 21: Regio infratemporalis, 7 and 22: Regio zygomatica, 8 and 23: Regio infraorbitalis, 9 and 24: Regio parotideomasseterica, 10 and 25: Regio buccalis, 11 and 26: Regio oralis, 12 and 27: Regio mentalis, 13 and 28: Fossa retromandibularis, 14 and 29: Regio mastoidea, 15 and 30: Regio occipitalis (according to Radlanski and Wesker (12), modified)

The clinical records of 179 patients with NF1 were analyzed. The patients were treated by the senior author for FPNF between 2000 and 2019. All patients met the current diagnostic criteria of NF1 [11]. The data were determined from the medical files, operation reports, histological findings, and doctor's letters and digitally recorded in an anonymous form. In addition, photograph documentation of the local findings of 112 patients from the archive of the senior author could be evaluated. Photographs of the patients were available taken before the procedure. In addition, intraoperative photographic documents of the tumor reductions were evaluated to verify tumor extension. Furthermore, magnetic resonance images of the patients were evaluated to verify the visually estimated tumor extension. The extent of the tumor thus assessed was manually transferred to a digitalized facial scheme [12] in every single case (Figs. 1 and 2). This individually recorded digital finding of tumor spread was merged into a single file with the recorded drawings of tumor spread from the other patients, which were archived in the same way. The software is able to calculate and scale accumulations of marked facial areas (as a feature of tumor spread) within this overall file. The computerized scaling is converted into arbitrarily selected color values, which then show the accumulation of the surgically treated regions at a glance. The schematic reproduction thus indicates the outline of the entire tumor that has been surgically treated. Since the focus of the study was on the surgical treatment of the patients, untreated tumors in the study area are not part of the registration. However, the unilateral and single-tumor manifestation is the typical expression of FPNF.

**Fig. 3:**
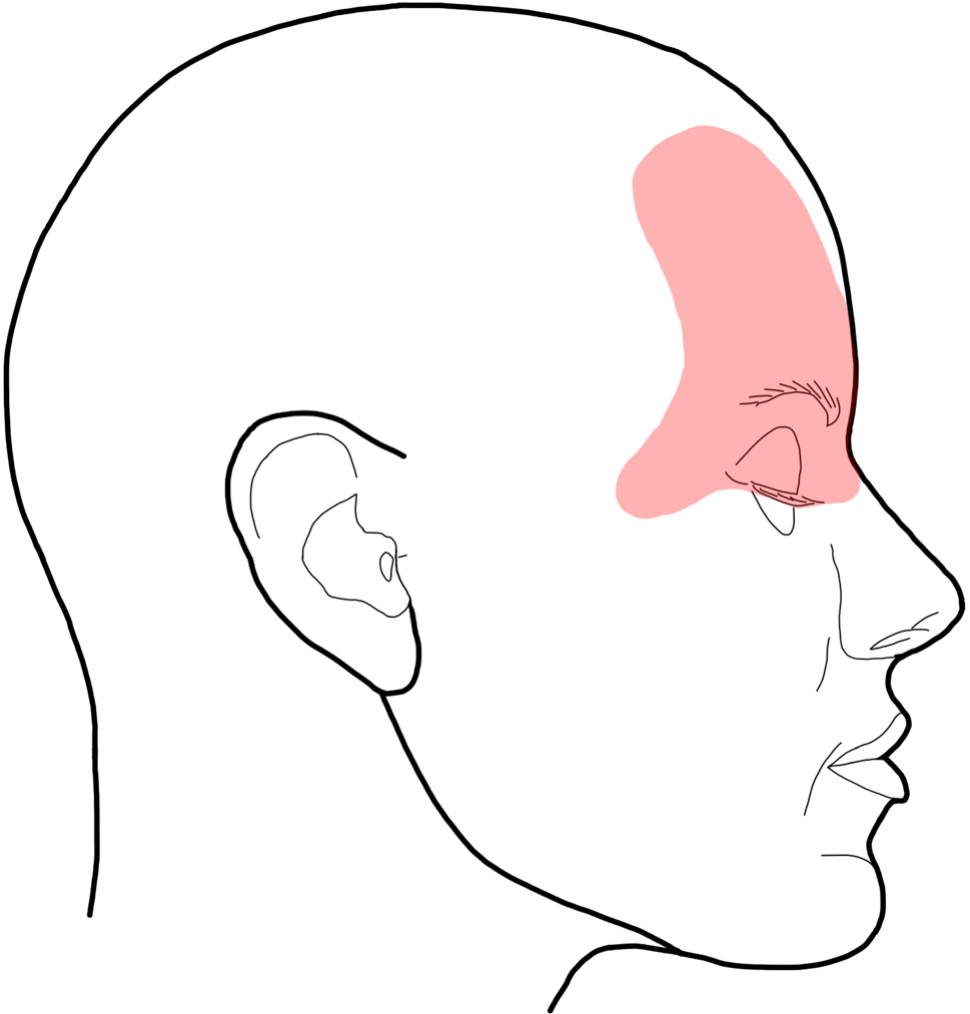
Illustration of the schematically reproduced right lateral aspect of the face with plotted right fronto-orbital tumor region (light red)

The digital files of tumor spread were documented in Photoshop™ Version 2.5 (Adobe; Mountain View, CA, USA). Two sets of images, each modified for and adapted to our investigations, each with four views (frontal, occipital, lateral right, and left) were prepared for each patient. The two sets of figures show white lines dividing the head into units or regions. The scaling of the skin surface in one set of images is based on the dermatomes of the examination area, the second on the defined anatomical units of the face and head. However, the initial recording of the tumor spread was made using a scheme without scaling in order not to influence the description of the tumor spread by pre-structured diagnostic fields. For each patient with existing photograph documentation, the respective extent of the tumor was drawn manually on the digital scheme (Fig. 3). This documentation resulted in different numbers with illustrations of the marked extent of the tumor (frontal: 108, occipital: 20, lateral right: 60, lateral left: 62).

The accumulation of the number of tumor localizations was illustrated with a heat map. More commonly affected/treated regions are illustrated in red, less commonly in yellow, and rarely in green. The duration of the operations was divided into 10-min increments (scale: 1–36) for a simplified overview of the data.

### Ethics

All procedures performed in this study involving human participants were performed following the ethical standards of the institutional and/or national research committee and with the 1964 Declaration of Helsinki and its later amendments or comparable ethical standards. Before analysis, data were anonymized, and the investigators studying the radiographs were blinded to diagnosis and individual identity. The investigations into anonymized data were performed following Hamburgisches Gesundheitsdienstgesetz (Hamburg Health Services Act). This type of investigation does not require the approval of the local ethics committee. The examinations are part of a scientific thesis to fulfill the requirements for the attainment of a doctorate at the faculty of medicine of the university (MM).

### Statistics

The statistical evaluation of the clinical data was carried out using SPSS version 27 (IBM, Armonk, USA). Absolute and relative frequencies, minimum, maximum, median, mean value (MV), and standard deviation (SD) were determined. The data were checked for normal distribution (Shapiro–Wilk test) before determining correlation coefficients. Correlations were calculated according to Spearman. Cases with an initially uncertain estimation of facial tumor spread from the documentation were jointly determined with the aid of written documents and diagnostic tools, i.e., photographs, surgical reports, and imaging (magnetic resonance images, computed tomograms).

## Results

### Diagnostics and Treatment

**Fig. 4:**
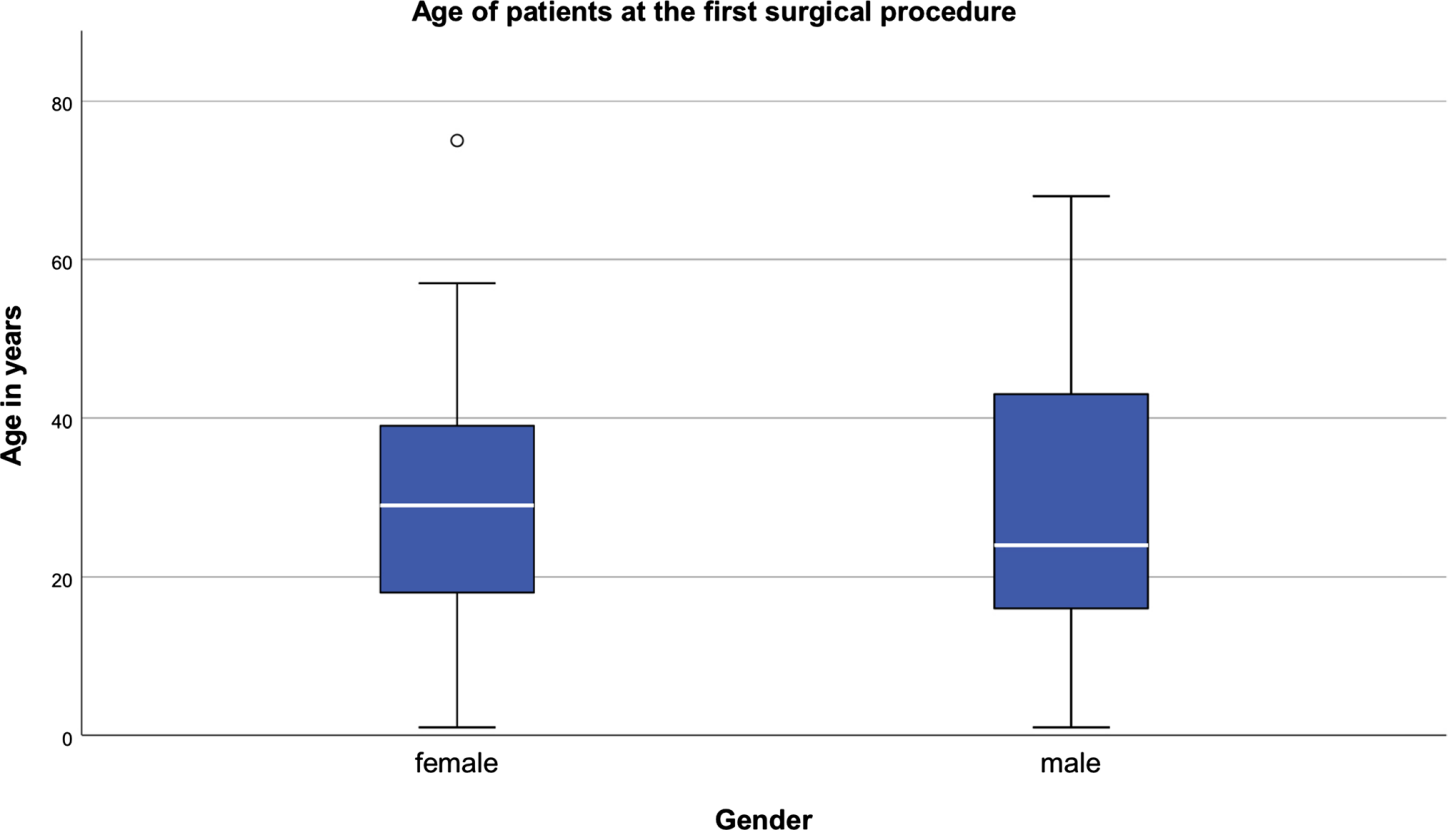
Age of patients at first surgical procedure in the facial region

Data of 105 female (58.7%) and 74 male (41.3%) NF1 patients were evaluated. The age at the time of the first operation varied considerably (females: 1–75 years, MV 29.24 ys, SD: 15.9; males: 1–68 years (ys), MV: 28.81 ys, SD: 15.98) (Fig. 4).

**Fig. 5:**
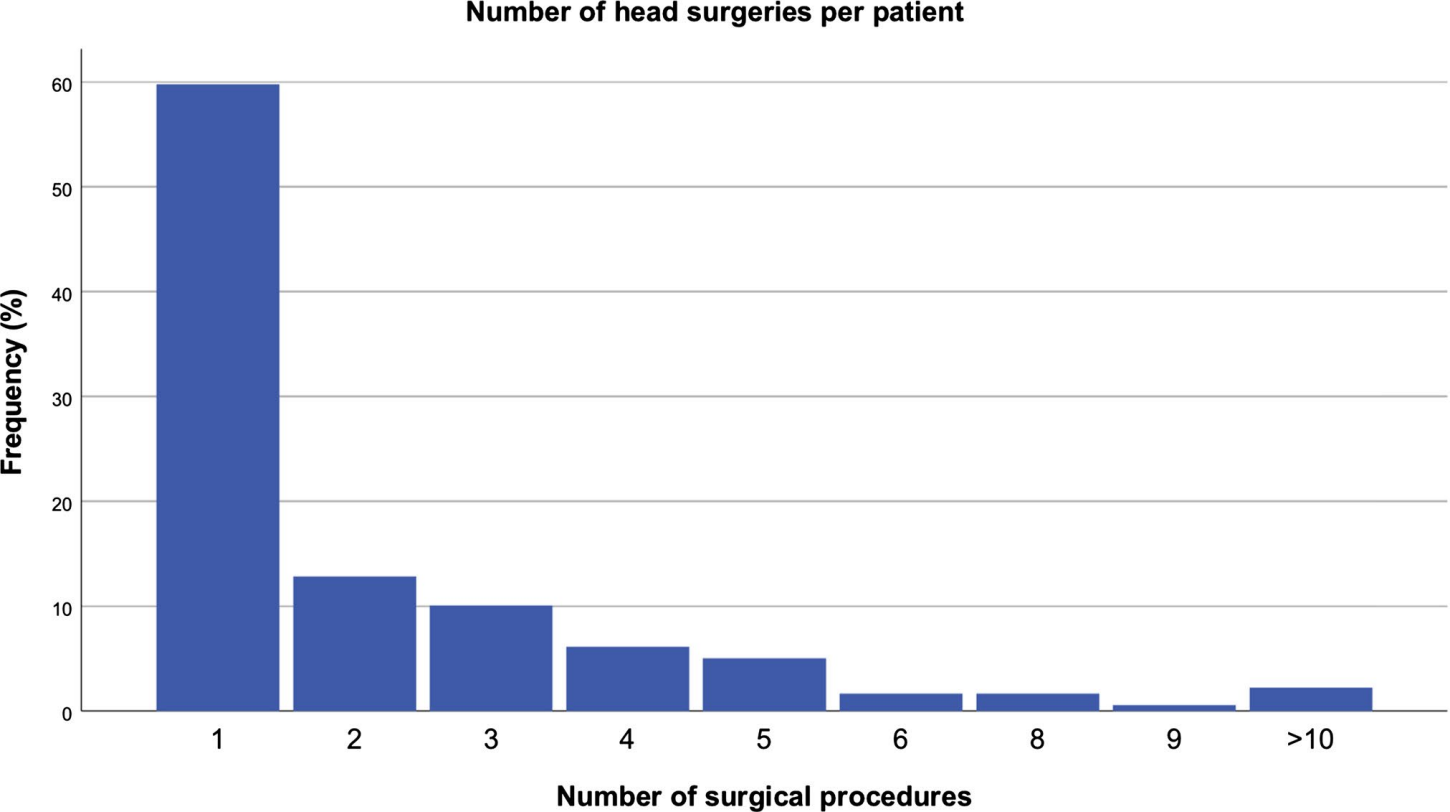
Number of surgical procedures

In the 179 patients, 395 surgical interventions were performed in the study area. The mean number of procedures per patient was 2.21 (SD 2.18, median: 1; minimum: 1, maximum: 13) (Fig. 5).

**Fig. 6:**
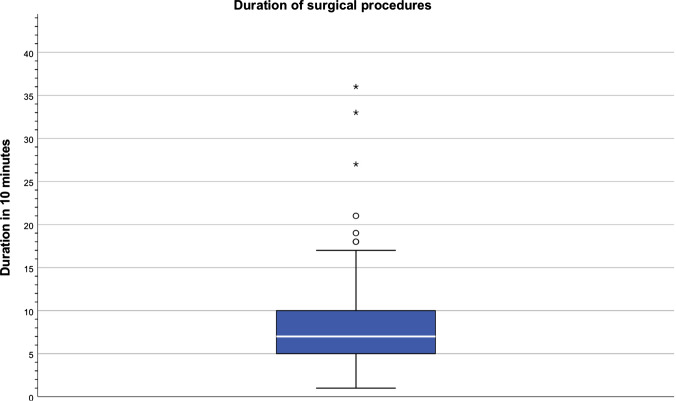
Duration of surgical procedures (10 min make one unit)

MV of surgery duration was 61–70 min (minimum: < 10, maximum: 351–360 min). Figure 6 illustrates the cumulated records.

**Fig. 7:**
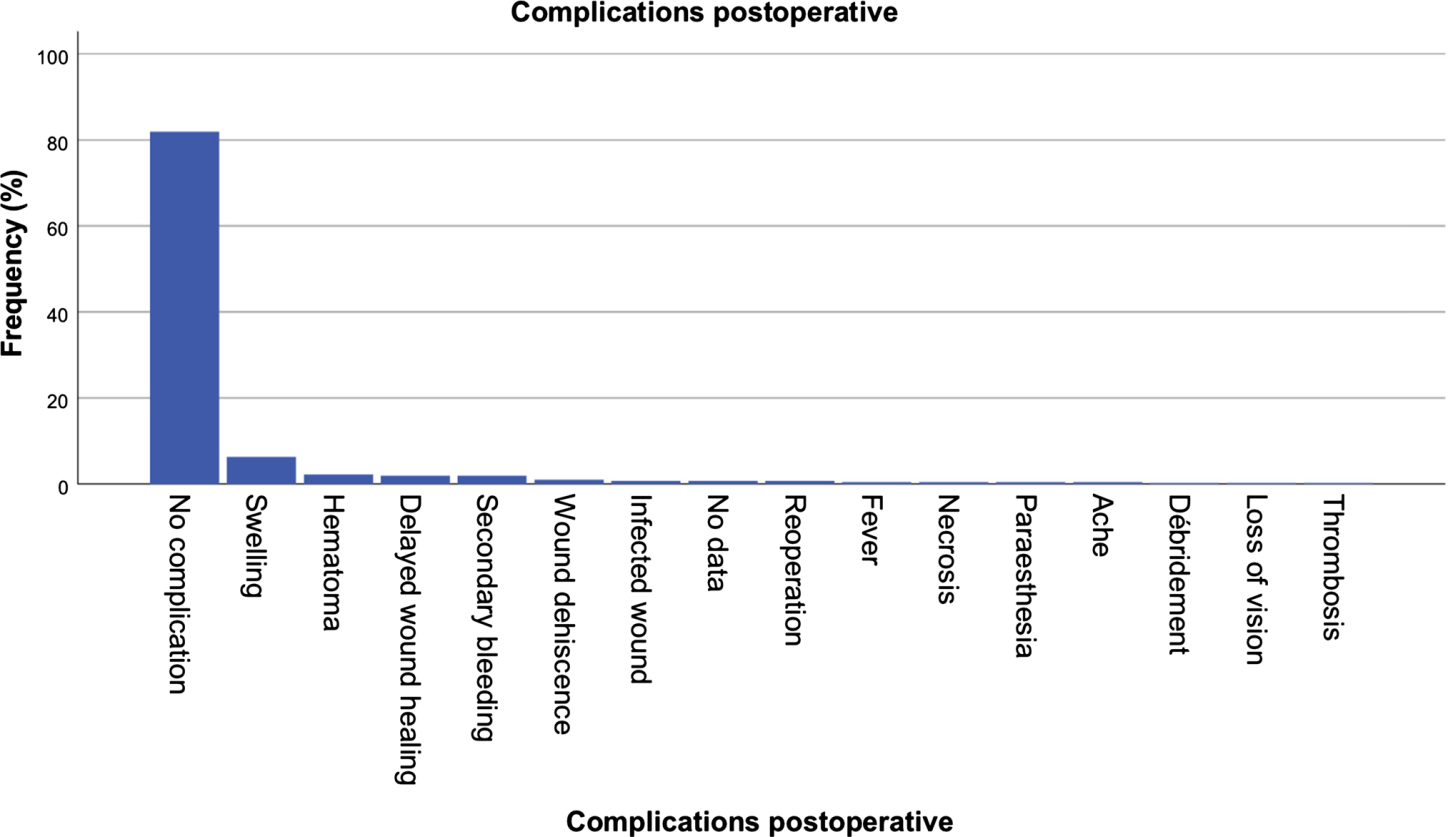
Complications following surgery

Relevant complications were not observed in most patients (81.9%). The main complications related to the interventions were unusually severe, extensive swelling, postoperative bleeding, wound dehiscence, and delayed wound healing (Fig. 7).

**Fig. 8:**
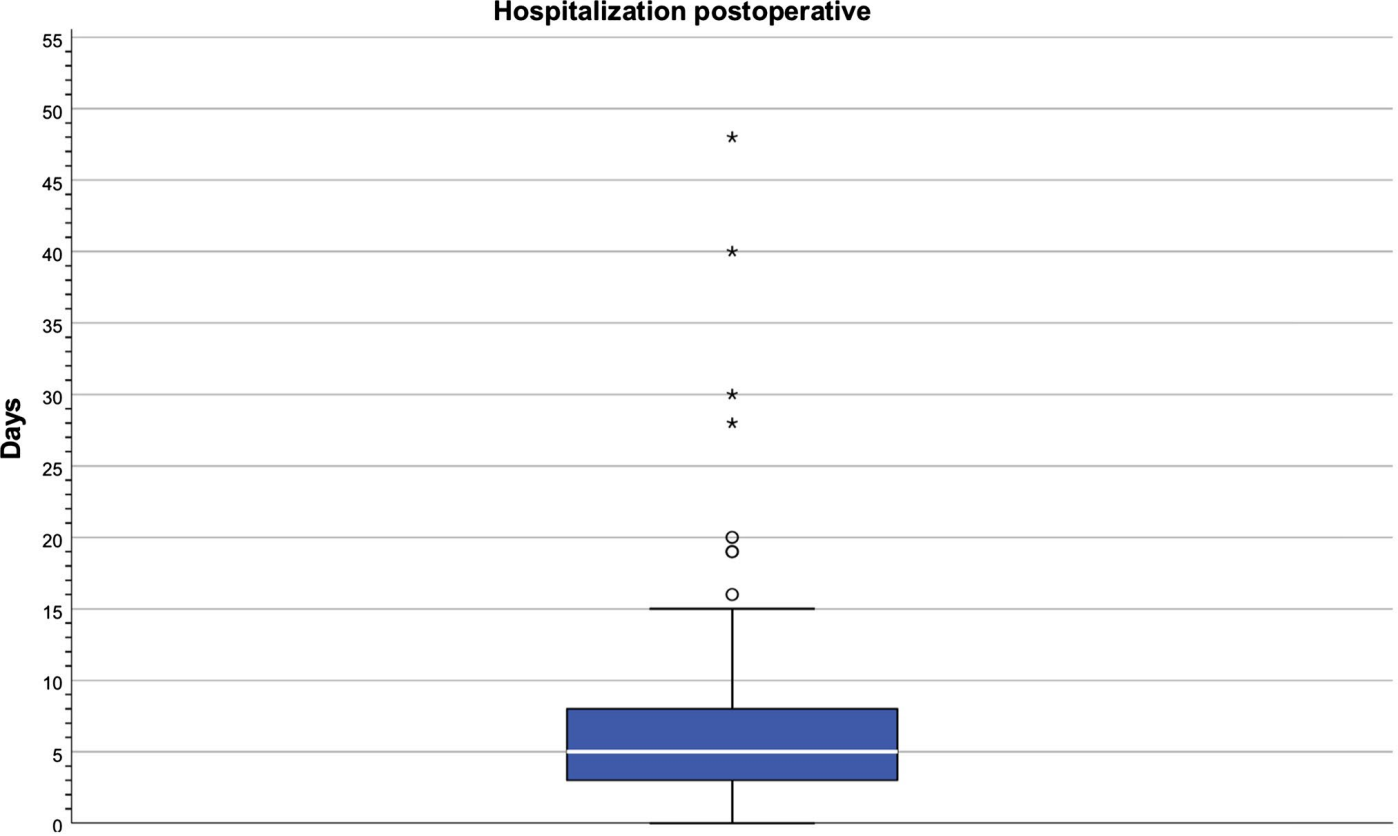
Inpatient treatment duration of NF1 patients

MV of the inpatient treatment duration was 6.24 days (SD 4.85 days, median: 5 days; minimum: = 0, maximum: 48 days). The length of hospital stays correlates with the number of dermatomes (coefficient: 0.261, *p* < 0.0001) (Fig. 8).

**Fig. 9:**
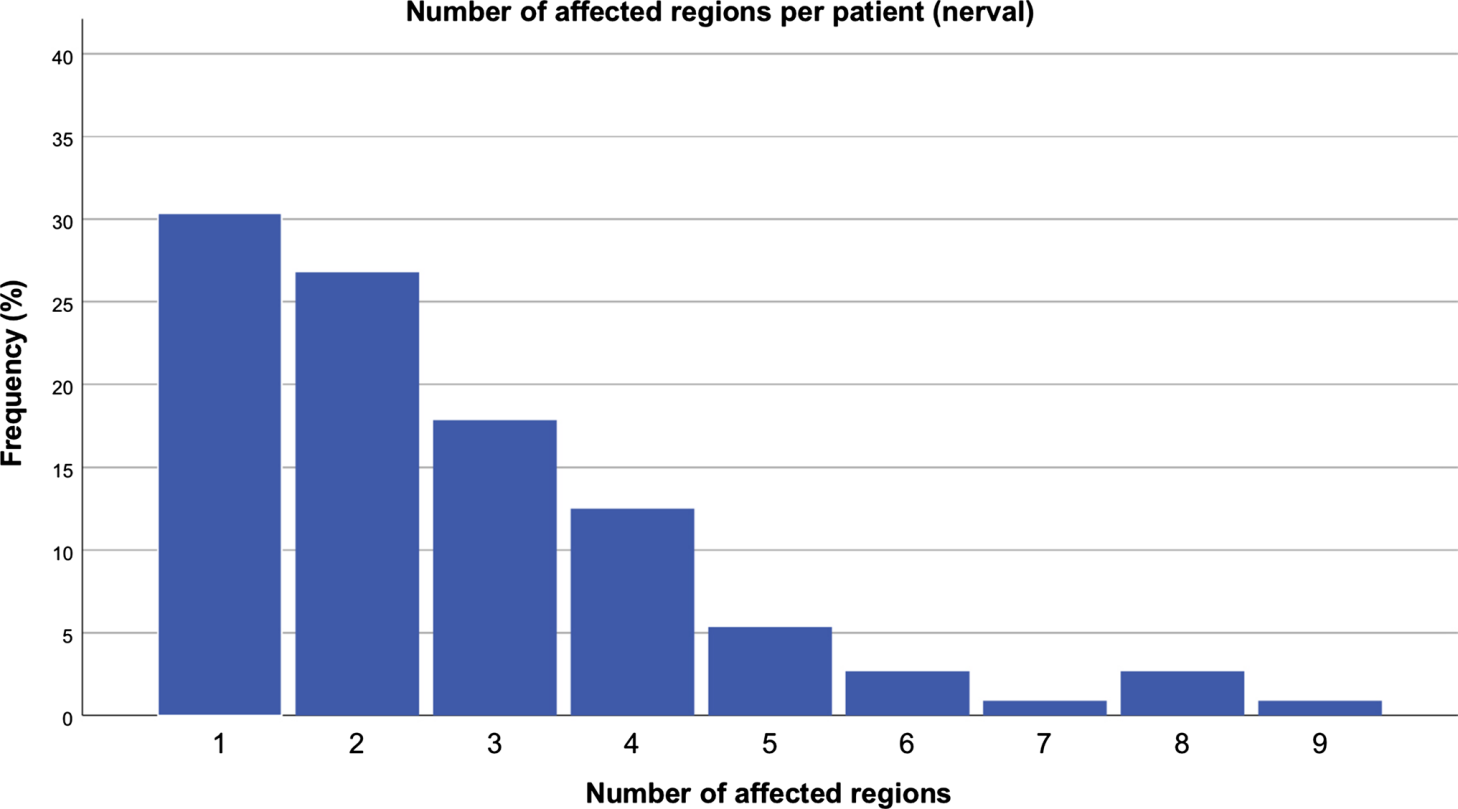
Number of affected regions (neural, dermatomes) (n = 112 patients)

The evaluation of the tumor localization of 112 patients showed 299 affected regions Table 1. More than half of the cases had tumors infiltrating one or two dermatomes (Fig. 9). The assignment of tumors to *dermatomes* showed most frequently affected the regions of the first trigeminal branch, followed by the two more caudally innervating branches. The frequency of other PNST in territories of other nerves accounted for 10% or less of the total number of regions (Table 2). The number of surgical procedures and the number of dermatomes correlate significantly (correlation coefficient: 0.340, *p* < 0.001). There is a correlation between the number of operations or the length of hospital stay with the number of tumor-affected dermatomes (*p* < 0.001).

**Fig. 10:**
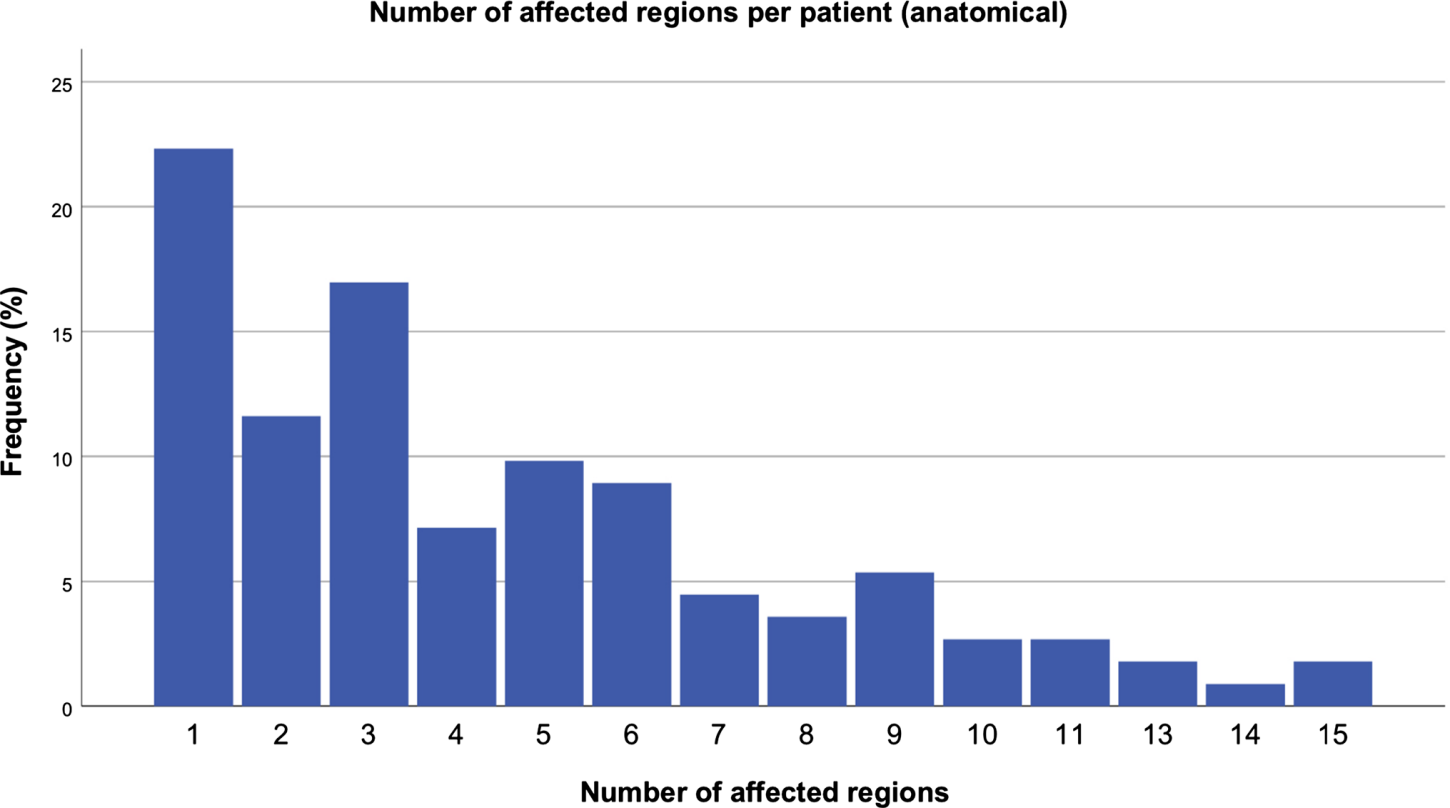
Number of affected regions (anatomical units) (n = 112 patients)

Subdivision of tumor extent by *anatomical* units confirms the preference for the orbital region, followed by the frontal and temporal regions (Fig. 10). Midface and lower facial regions follow in descending order of frequent tumor locations. The relatively rare occurrence of FPNF in the infraorbital region compared to the orbital region is striking. However, according to anatomical classification, the units of the face are equally affected over a wide area (Table 2).

### Heatmap

**Fig. 11:**
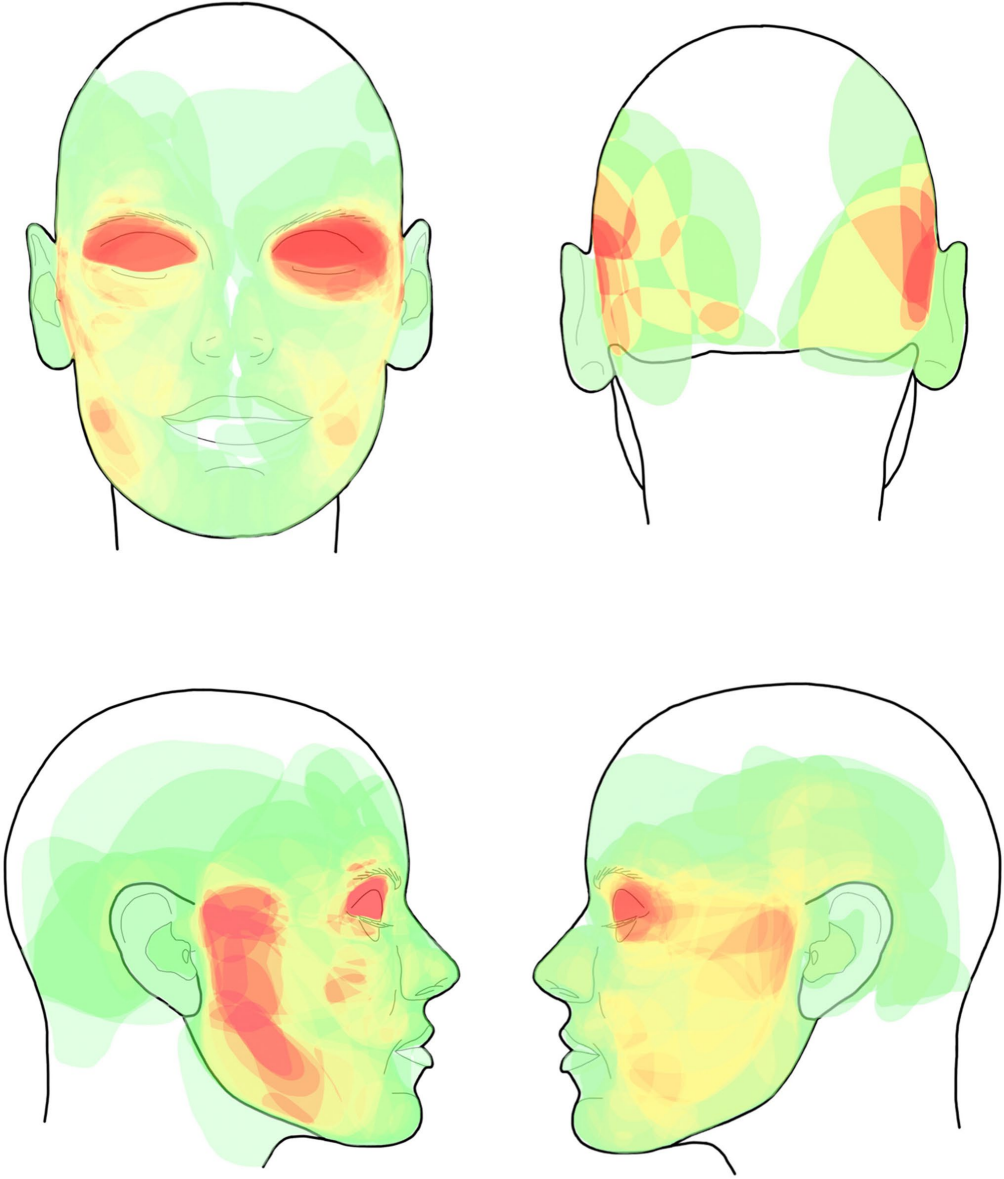
Color-coded distribution frequency (heat map) of tumor spreading in the facial region of NF1 patients with facial plexiform neurofibromas (FPNF). Especially the eyelids (tumor reduction/ptosis surgery) and the cheek/temporal region (face lifting procedures) were affected

The distribution of the tumors in the examination area was illustrated using a heat map (Fig. 11). Classification of tumor frequency by dermatome shows that more than 50% of patients were affected in one or two dermatomes. High numbers of dermatome counts are all below 20%. On the other hand, the distribution of the tumors according to anatomical units differentiated much more finely than the dermatomal allocation. In the former, tumors with one or two affected regions reach just under 34% of the population. Patients with 3–8 affected units represent more than 50% of the patients. The number of tumor-affected dermatomes correlates with the length of hospital stay.

## Discussion

The study analyzes the treatment data of surgical measures of patients with FPNF. The study applies an overlay technique to describe the frequency of tumor localization. The study results provide an overview of facial regions in particular need of treatment. Furthermore, clinical treatment data are analyzed to specify inpatient treatment needs. The presented quantitative analyses of treatment data provide a guide for planning facial surgical procedures in patients with FPNF. However, in individual cases, clinical treatment data may differ from the values presented here if unusual tumor manifestations are included in the treatment calculation. The complication rate is just under 20% and justifies the inpatient care of the patients as well as the length of stay in the hospital individually adapted to the course of treatment. The correlation analysis shows the relationship between the number of dermatomes affected and the number of surgeries and hospital days of stay after surgery.

### Clinical Data

Topographic analysis of craniofacial PNF tumor spreads is an important contribution to assessing the treatment needs of these patients. Among NF1-associated PNF, craniofacial PNF requires the most surgical treatment [3, 13]. Both classifications of the facial surface used for topographic evaluation show PNST frequency symmetry illustrated by the tumor-affected regions marked with the same intensity of color on both sides, namely eyes/orbits, temples, cheeks, and lateral forehead. The distribution pattern confirms the assumption that there is no side predilection for the tumor type. The need for surgical treatment focuses on the orbital and periorbital regions and face lifting procedures in cases with extensive tumor spread. However, it must be considered that the determination of tumor topography was performed using predominantly surgical photographs, and therefore the exact tumor spread may be more extensive than the external visual appearance or data evaluation suggests. Classification of tumor locations according to anatomical regions identifies tumor growth extending beyond single regions. According to the present study, the association of tumors with anatomical regions is an uncertain indicator of tumor spread and load, both because tumor spread does not respect these boundaries and because tumor infiltration can destroy these landmarks. However, this finding should not be generalized to the entirety of FPNF. A bias in the NF1-associated size of facial tumor extensions in favor of more advanced and extensive manifestations in a reference center for the treatment of NF1 patients is very likely. In other words, smaller, circumscribed tumors of this type can be reliably assigned to an anatomic region. Superimposing the affected region with anatomical landmarks, e.g., by mirroring the unaffected side of the face onto the diseased side, is a practical step in the assessment of tumor-associated changes in hard and soft tissues. This procedure is based on the fact that the neural territories of tumor-altered facial regions do not cross the anatomical boundary, i.e., the median-sagittal plane [14]. The classification of the facial skin surface according to dermatomes shows that in individual cases, the tumors can be assigned very variably to the defined areas. There are cases in which the tumors infiltrate the dermatome only partially or grow beyond the borders according to visual inspection. On the other hand, overlapping of tumor-invaded facial dermatomes is frequent, so classification must be based on combinations of adjacent dermatomes with tumor-related skin changes. Nevertheless, orientation to the dermatomes is an essential and reliable clue in defining facial tumor spread and associated skeletal lesions [8, 15]. In this respect, there is a difference between the assignment of the FPNF spread of NF1 patients to the extremities and dermatomes. In these regions, the segmental distribution of tumors across body regions was obvious [9, 10]. The two-dimensional evaluation method chosen here is suitable for assessing the individual course of the disease (e.g., to objectify disproportional tumor growth) and planning surgical interventions in the facial regions.

The evaluation of histological findings illustrates consensus between clinical assessment and tissue findings in many cases [16]. However, a review of tissue findings reveals that clinical assessment of misshapen, often large skin tumor regions according to histologic criteria encompasses different subtypes of PNST. The difference between the clinical assessment of a large PNST as "plexiform" and histological diagnosis of these tumors mainly concerns the distinction between PNF, diffuse neurofibroma, and diffuse PNF (Table 2). In a recent Japanese study, the distribution of PNF on the body surface was mapped [17]. A total of 19.2% of diffuse PNF were in the head and neck region. However, the cartography of the tumors was not classified according to dermatomes. Likewise, the diagnosis of PNF was not further specified. Other studies suggest that craniofacial PNF is among the most common tumor sites in NF1 patients requiring surgical intervention [3, 13]. However, from clinical judgment, the differences between PNF and diffuse neurofibroma appear to be gradual (Table 2).

**Table 1:** The frequency of affected regions of the face and adjacent regions. Evaluation of all affected regions of the corresponding image series with view from frontal, occipital, right (R) and left (L) lateral. The tumors were assigned according to anatomical units on the one hand, and according to dermatomes of the head on the other hand. Data of the frequencies of tumor localizations in absolute values and percent are listed in descending order (N = Nerve)

Affected anatomical units (Region, Fossa): Number (%)	Affected dermatomes: N (%)
Regio orbitalis (L): 39 (7.7)	N. ophthalmicus (L): 42 (14.0)
Regio orbitalis (R): 37 (7.3)	N. ophthalmicus (R): 41 (13.7)
Regio frontalis (L): 27 (5.3)	N. maxillaris (L): 38 (12.7)
Regio temporalis (L): 27 (5.3)	N. maxillaris (R): 30 (10.0)
Regio frontalis (R): 25 (4.9)	N. mandibularis (L): 30 (10.0)
Regio infratemporalis (R): 23 (4.5)	N. mandibularis (R): 29 (9.7)
Regio temporalis (R):22 (4.3)	N. auricularis magnus (R): 16 (5.4)
Regio infratemporalis (L): 22 (4.3)	N. occipitalis minor (L): 16 (5.4)
Regio zygomatica (L): 22 (4.3)	N. auricularis magnus (L): 14 (4.7)
Regio zygomatica (R): 22 (4.3)	N. mandibularis/N. maxillaris (L): 10 (3.3)
Regio oralis (L): 22 (4.3)	N. occipitalis major (L): 9 (3.0)
Regio parotideomasseterica (R): 21 (4.1)	N. occipitalis minor (R): 8 (2.7)
Regio buccalis (R): 21 (4.1)	N. mandibularis/N. maxillaris (R): 6 (2.0)
Regio buccalis (L): 20 (3.9)	N. occipitalis major (R): 5 (1.7)
Regio parotideomasseterica (L): 19 (3.7)	N. mandibularis/N. ophthalmicus (R): 2 (0.7)
Regio oralis (R): 17 (3.3)	N. mandibularis/N. ophthalmicus (L): 2 (0.7)
Regio infraorbitalis (R): 17 (3.3)	N. occipitalis major/N. ophthalmicus (R): 1 (0.3)*
Regio infraorbitalis (L): 16 (3.1)	
Regio nasalis (R): 14 (2.8)	
Regio nasalis (L): 11 (2.2)	
Regio parietalis (L): 9 (1.8)	
Regio occipitalis (L): 9 (1.8)	
Regio mentalis (R): 9 (1.8)	
Regio mentalis (L): 8 (1.6)	
Regio occipitalis (R): 7 (1.4)	
Regio parietalis (R): 5 (1.0)	
Regio mastoidea (R): 5 (1.0)	
Regio mastoidea (L): 5 (1.0)	
Fossa retromandibularis (R): 4 (0.8)	
Fossa retromandibularis (L): 3 (0.6)	

^*^ No case with right-sided combination of tumor spread in the dermatomes of these nerves

**Table 2:** Histological findings of surgical specimen

Histology	Frequency (N/%)
Diffuse neurofibroma	189 (35.5)
PNF	120 (22.5)
Diffuse plexiform neurofibroma	85 (15.9)
Insufficient data	63 (11.8)
Cutaneous neurofibroma	25 (4.7)
Neurofibroma, not further specified	23 (4.3)
Atypical neurofibroma	9 (1.7)
MPNST	7 (1.3)
Neuroma	2 (0.4)
Neurofibroma with Schwannomatous differentiation	2 (0.4)
Nevus	2 (0.4)
Benign PNST	1 (0.2)
Precursor cutaneous neurofibroma	1 (0.2)
Irritant fibroma	1 (0.2)
Verruca vulgaris	1 (0.2)
Foreign-body granuloma	1 (0.2)
Giant cell granuloma	1 (0.2)
Total	533 (100)

### Complications

Postoperative bleeding must be considered in the reduction of plexiform neurofibroma. Bleeding can already influence the extent and duration of the measures during the surgical intervention [18]. However, the wound healing results are aesthetically satisfactory [19].

### Graphic Overlay Technique (Heat Map) of the Affected Facial Regions

The superimposition of facial findings on digital schematics with the outlines of the head or face is currently a widely used diagnostic tool for illustrating local, visible findings (e.g., tumors or malformations of the facial skin), including the assessment of their progression [20, 21]. Furthermore, digital facial images are used to study myocutaneous functions [22] and the expressive behavior of defined examination groups based on the automated recognition of facial landmarks [23]. Determining the frequency of nosologically defined facial findings concerning anatomical regions can support the characterization of predilection sites for a disease [20].

The approach was employed in the current investigation to identify the therapy requirements for a group of individuals with a tumor predisposition syndrome. Tumors of the face that are distinctive and frequently deforming may manifest in NF1. The selection criterion allows for an assessment of which regions are particularly commonly examined by patients needing treatment, but it does not allow for safe conclusions to be taken regarding the incidence of certain regions of the face acquiring PNF in the community of NF1 patients.

Individual color-coded overlays of the frequency distribution of PNF-related craniofacial skeletal dysmorphology [24] and soft tissue abnormalities [21] should aid in a better understanding of the complicated pathology of the area, possible treatments, and outcomes.

Additionally, the simultaneous surface representation of bone and skin in a projection should be useful for planning combined skeletal-soft tissue surgeries on the craniofacial region in NF1-affected patients.

### Limitations of Study

The study has several limitations. On the one hand, the borders of the tumor that are visible or defined by tools (imaging, surgical documentation, and histology) were accepted as the true extent of the tumor and transferred to the diagram. Inaccuracies in the registration and in the transmission may have had an impact on the outline of the registered area in individual cases. However, this inaccuracy mainly affects the edges of the tumor and therefore probably has little effect on displaying main fields of tumor spread visible as the superimpositions of the individual tumor extensions. A systematic limitation of the procedure is the fundamental uncertainty of the extent of the tumor in the individual case. However, this criticism applies to every clinical study on the subject. Because many tumors could not be completely removed, histologic confirmation of tumor margins determined by visual assessment of the situs and evaluation of imaging is lacking. Furthermore, diffuse PNF in particular disperse very thinly in the edge areas of the lesion, so that only the histological examination can confirm the quality of the resection edge. However, such thin and subcutaneously infiltrating, inconspicuous tumors are less often the target of surgical interventions. Furthermore, this is a cross-sectional study on a nosologically defined collective. NF1 is a chronic progressive disease. Although facial PNF are regarded as congenitally manifest tumors, in our own experience the potential for growth and infiltration does not end at the end of the growth period. The visual definition of the tumor boundaries of the cross-sectional study reflects only the graphically simplified current extent of tumor spread at a certain point in time. In individual cases, a follow-up study using this graphic technique would offer an abstract assessment of tumor growth and derived from this in clinical studies on larger patient groups, information on the preferred direction of growth and the expected tumor volume.

## Conclusion

The studies show that surgical treatment of FPNF of NF1 patients is associated with relatively few complications in the majority of patients, which can be safely treated in a surgical clinic. However, these are palliative measures to improve the quality of life in patients with chronic progressive disease and often locally destructive tumors. The surgical approach should incorporate the need for repeated procedures. This advice should already be considered in the surgical consultation. Digital processing of photographic documents of tumor spread allows rapid assessment of main fields of surgical interventions through the preparation of a heat map: In this study, the need for surgical action in a larger group of patients was clearly illustrated by the heat map and can be judged at a glance. Other potential applications in the field of PNF surgery include assessments of tumor reduction and recurrence following surgery, visualization of the influence of tumor biology on shape and volume in the affected part of the body in untreated cases over time (for example, the influence of both invasive tumor growth in the area and sagging of the tumor-infiltrated connective tissue on the outline of the tumor), but also as monitoring of the effects of the recently introduced drug therapy of symptomatic, inoperable PNF on tumor volume.
